# A Mixed Methods Service Evaluation of a Peer-Supported Breakfast Group in Adult Inpatient Burn Rehabilitation

**DOI:** 10.3390/ebj7030040

**Published:** 2026-07-17

**Authors:** Lottie Elizabeth Armitage

**Affiliations:** 1 School of Health and Social Wellbeing, University of the West of England, Bristol BS16 1QY, UK; lottiearmitage@nhs.net; 2 St. Andrews Centre for Burns and Plastic Surgery, Broomfield Hospital, Mid and South Essex NHS Foundation Trust, Chelmsford CM1 7ET, UK

**Keywords:** rehabilitation, peer support, occupational therapy, burn injuries

## Abstract

**Highlights:**

**What are the main findings?**

- Human connection and shared experiences emerged as a central benefit, reducing isolation and increasing psychological safety.
- Routine building, autonomy and functional practice were consistently supported, with the non-ward environment enhancing motivation, confidence and ”real-world” simulation.

**What are the implications of the main findings?**

- Breakfast groups may be a valuable component of inpatient burn rehabilitation, offering functional and psychosocial benefits beyond standard individualised therapies.
- Structured opportunities for peer connection could support reduced isolation and optimisation of emotional wellbeing and enhance rehabilitation engagement.

**Abstract:**

**Background**: Burn injuries have complex physical and psychological consequences, making holistic rehabilitation essential. This mixed methods service evaluation explored the acceptability and perceived benefits of a peer-supported breakfast group delivered as part of routine occupational therapy practice for adult inpatient burn survivors. **Methods**: A tailored survey integrating PROMIS items with open-ended questions was completed by nine participants. All English-speaking inpatients aged >18 years who attended were invited to participate. In total 36 patients attended and 9 completed the survey (*n* = 36; *n* = 9). A convergent design integrated open-ended survey responses with PROMIS patient-reported outcome items, analysed thematically and interpreted using the RE-AIM (Reach, Effectiveness, Adoption, Implementation, Maintenance) and MMR-RHS (Mixed Methods Reporting—Rehabilitation and Health Sciences) frameworks. **Results**: Quantitative findings suggested high perceived physical function and strong emotional support, with low anxiety and depression, and low social isolation. Reflexive thematic analysis generated four themes: fostering human connection and emotional wellbeing; restoring autonomy and confidence; preparing physically and psychologically for discharge; and the influence of the rehabilitation environment. **Conclusion**: While descriptive only, findings provide early insight into the value of peer-supported group activity in inpatient burn care. Larger controlled studies with baseline measurement are needed to evaluate effectiveness and implementation feasibility.

## 1. Introduction

Burn injuries are multifaceted, causing physical harm alongside profound psychosocial consequences that can hinder an individual’s capacity to engage in daily activities and fulfil social roles [1,2]. Addressing this requires comprehensive rehabilitation, prioritising both functional recovery and psychosocial wellbeing [2,3].

Survivors can experience post-traumatic stress disorder (PTSD), depression, anxiety, stigma, and body image concerns [1,2], with difficulties often persisting long after discharge and influencing reintegration into family, social, and occupational life. Physical impairments—including chronic pain, fatigue, and the development of contractures, which restrict mobility and limit independence—can further compound this [2,4,5]. These physical and psychological components interact and intensify each other.

Social participation is commonly disrupted, with survivors facing difficulty in returning to work or community activities, due to the combined physical and psychosocial barriers [6]. Peer support has been shown to strengthen psychosocial resilience, reduce isolation and foster belonging by enabling survivors to share and validate experiences [6,7,8]. Despite evidence from burn camps and support groups highlighting these values, peer-supported approaches remain underutilised in inpatient burn care settings.

Therapeutic group programmes can facilitate a sense of normalcy, foster motivation, enhance self-efficacy and social confidence, supporting reintegration following trauma [6,7,8]. Yet survivors report feeling inadequately equipped when psychosocial recovery is overlooked in favour of physical recovery [9], highlighting the significant potential of group-based therapeutic approaches in fostering a more sustainable and holistic recovery [10,11].

Occupational therapy groups operate along a spectrum ranging from support groups to activity-based groups, focusing on targeting functional skills [12]. Despite differing emphases, these groups offer overlapping benefits. Activity-based interventions can improve movement, quality of life, and social interactions [13,14,15,16], and ensuring this is meaningful to individuals can support psychosocial recovery and aid smoother transitions between hospital and home [13,17]. Group-based interventions may further offer cost-effective rehabilitation strategies [14].

Evidence for group-based interventions in inpatient burn populations remains limited, with only one published study evaluating a cooking group within this context [16]. Given the potential advantages of such an intervention and the gap in current research, addressing this issue is both timely and necessary.

In 2022, an occupational therapy-led breakfast group was established on the acute adult rehabilitation ward to enable inpatient survivors to come together to both prepare and eat breakfast as part of an occupational therapy-based activity. The proposed benefit was to engage rehabilitation across multiple domains—physical, cognitive, and psychological—and enable the acquisition of daily life skills alongside providing routine and normalcy.

This service evaluation explored the acceptability, perceived benefits, and feasibility of the breakfast group as part of routine inpatient burn rehabilitation. Specifically, it aimed:

- To explore survivor-reported experiences of participation in social roles and activities.
- To examine perceived influences on social connection and isolation.
- To describe perceived emotional wellbeing during inpatient recovery.
- To identify perceived functional benefits related to daily living activities and overall recovery.

## 2. Materials and Methods

### 2.1. Study Design

This was a convergent mixed methods service evaluation conducted in routine clinical practice at a single UK burn centre. This design was chosen to capture participant experiences of the group at a single time point rather than measure changes over time. Pre/post or repeated measures were not feasible within routine inpatient workflow, as participants attended with varying frequency and length of stay. Although attendance was routinely documented, survey anonymity prevented linking individual responses to attendance records, so a control group of non-attenders could not be identified. This design allowed experiential insights to be obtained whilst maintaining ethical and practical constraints of real-world rehabilitation.

Qualitative and quantitative data were collected concurrently and interpreted together to contexualise descriptive trends within the survivor narratives. Integration followed the Mixed Methods Reporting—Rehabilitation and Health Sciences (MMR-RHS) framework [18] and the RE-AIM (Reach, Effectiveness, Adoption, Implementation, Maintenance) framework [19] to consider perceived benefit and practical feasibility.

Participation reach was examined using open-ended questions about barriers, facilitators, and challenges, providing narrative insights into who engaged and why. Likert-scale items, including pain-interference questions, captured specific, measurable factors influencing participation. Perceived benefit (reported under ”effectiveness” within the RE-AIM framework) was assessed using complementary methods: open-ended questions explored motivation and perceived outcomes, while Likert-scale items measured symptoms and functional factors relevant to burn injury.

**Figure 1:**
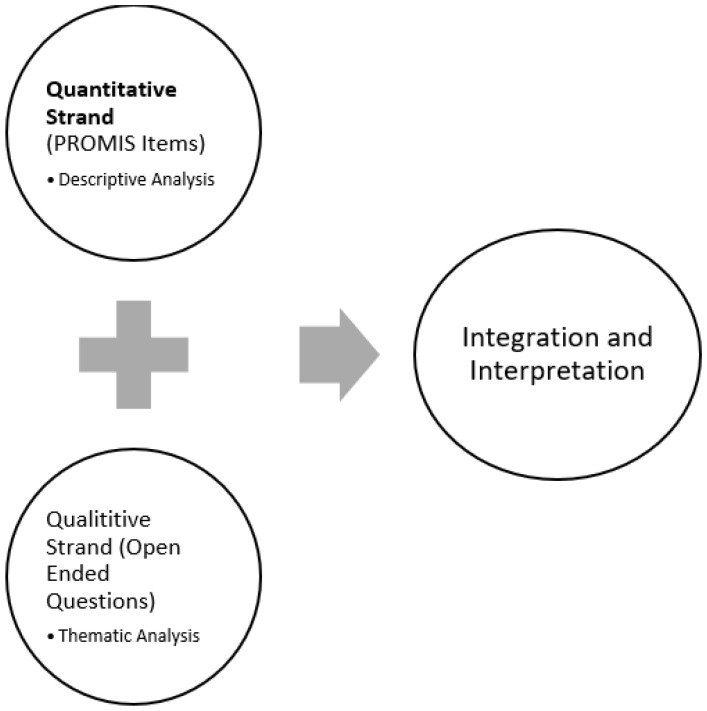
Visual summary of data integration. Plus signs represent merging of inputs, and the arrow shows the final synthesised outcome.

These factors are mutually informative since barriers such as pain may directly influence engagement and perceived benefit [3,9]. RE-AIM complemented MMR-RHS by offering a practical structure to integrate and report qualitative and quantitative findings clearly (Figure 1).

### 2.2. Indications for Inpatient Burn Rehabilitation

Inpatient burn rehabilitation within the UK healthcare system is provided to individuals requiring multidisciplinary input following burn injury. Eligibility typically includes patients with functional limitations affecting mobility, self-care, or participation in daily activities as well as those requiring psychological support to aid adjustment and recovery. These criteria contextualise the population from which group participants were drawn.

### 2.3. Eligibility for the Breakfast Group

Eligibility for the breakfast group was determined by clinical staff based on the ability to take oral nutrition, mental capacity to consent and the absence of infection-control restrictions. The group was delivered as part of a routine occupational therapy activity in the ward day room, offering opportunities to prepare simple breakfast items, practice daily living skills and eat together in a communal space. Participants attended between one and several sessions, depending on clinical status, discharge timing, and personal preference.

### 2.4. Study Inclusion and Exclusion Criteria

Purposive eligibility criteria were applied to ensure participants reflected the intended scope of the service-evaluation aims [20], with inclusion and exclusion criteria summarised in Table 1.

All English-speaking adults (>18 years) admitted to the adult burns ward who attended the breakfast group during the study period were eligible for invitation. Clinical criteria used to determine eligibility to attend the breakfast group (and therefore study invitation) included the ability to take oral nutrition, mental capacity to consent, and absence of active infection-control restrictions.

Recruitment was convenience-based, consistent with similar group-based evaluations [9,15]. Posters at the intervention location, template invitation letters, and direct approach by clinical staff during inpatient stay or outpatient follow-up were used. Invitations were issued between February and March 2025. The invitations were sent to all participants eligible for the breakfast group over the 2.5-year period who met the inclusion criteria for this project.

Of 36 invited, 11 responded; 2 declined participation citing emotional readiness and distress related to revisiting their injury experience. Nine returned completed surveys and were included in the final analysis (completion rate 25%).

**Figure 2:**
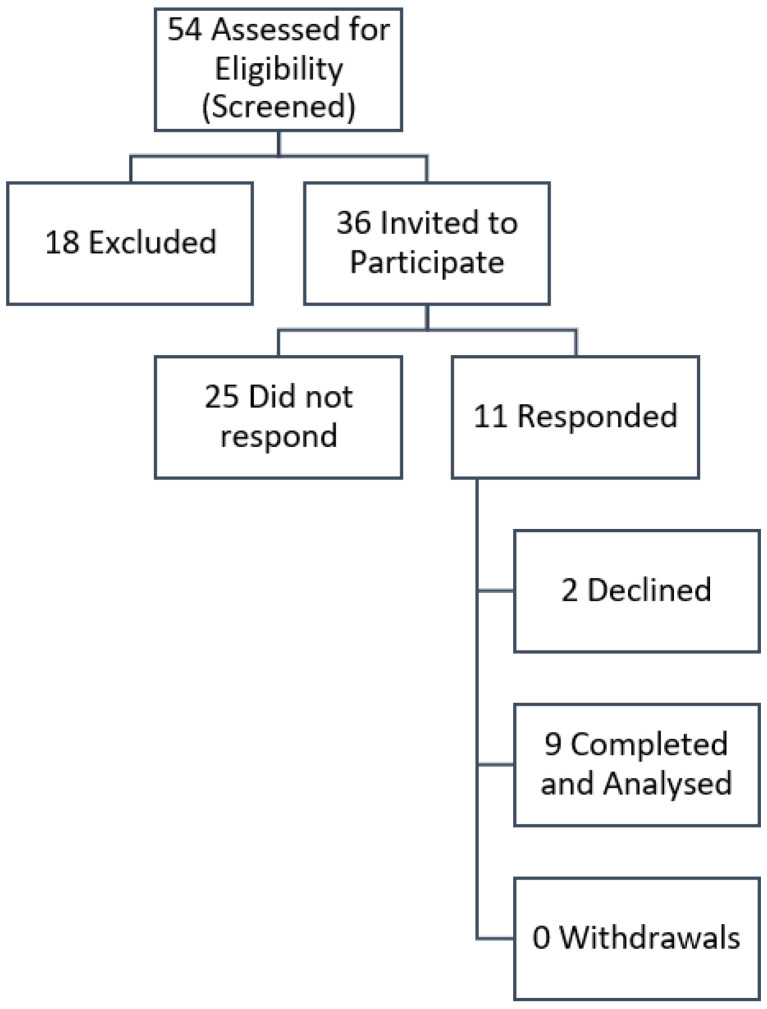
CONSORT-style participant flow.

A CONSORT-style participant flow diagram is provided in Figure 2. No formal sample-size calculation was performed; sampling was pragmatic and determined by the number of eligible inpatients invited during the recruitment window.

Demographic data for respondents were not collected due to the anonymous survey design.

### 2.5. Ethics

Although conducted within routine care, this project was reviewed and approved by institutional ethics (University of the West of England Ethical Review: 13506819) and audit committees. Participants provided informed written consent, and the study adhered to the principles outlined in the Declaration of Helsinki [21].

### 2.6. Data Collection

Survey responses were completed in written format, either on paper or electronically. No audio recordings were used—paper surveys were de-identified and stored securely before transcription into Microsoft Forms. Electronic responses were submitted directly into the institution’s secure cloud-based system. All data were collected anonymously, consistent with routine clinical governance procedures.

No validated measure exists to determine the specific effectiveness of group-based activity in burn-injured populations. Existing burn measures considered (SF-36, EQ-5D, and Burn Specific Health Scale) primarily assess individual factors and do not capture constructs relevant to group dynamics such as peer interaction or the influence of shared therapeutic environments. These tools lacked sensitivity for the group-specific psychosocial and physical outcomes, justifying a custom approach [22,23,24]. A tailored survey was developed to align with the evaluation aims and ensure sensitivity to the social, environmental and experiential aspects of the group (see Supplementary Materials).

The survey included twenty-one Likert-scale items selected from the Patient-Reported Outcomes Measurement Information System (PROMIS) subscale bank—pain interference, physical function, emotional distress (anxiety and depression), emotional support, and social isolation, as domains relevant to burn injury and group-based activity [2,3,9,16,25,26,27]. PROMIS items were analysed as individual 1–5 scores, with item-level means reported. This approach was chosen over T-scores given the small sample size and the need for direct, clinically interpretable results relevant to burn rehabilitation.

Five open-ended questions were included to obtain contextualised information about influences on participation and experience. Pilot testing was not feasible given time and population constraints. Methodological triangulation was used to strengthen validity [28,29]. This involved systematically comparing thematic patterns with the PROMIS domain scores to identify areas of convergence and divergence. The comparisons allowed quantitative trends to be interpreted alongside the survivor narratives that shaped these. This ensured that the interpretation remained survivor-centric with the quantitative data used to support, not replace, the survivors’ experiences.

Time elapsed between group attendance and survey completion varied due to differences in discharge timing and outpatient follow-up.

### 2.7. Data Analysis

Descriptive statistics were calculated using Microsoft Excel, including means, standard deviations, medians, interquartile ranges, ranges, and modes for each PROMIS item set. Participants rated items on a 1 (very poor) to 5 (excellent) scale. For scoring, higher values indicate better outcomes for physical function and emotional support, with higher values indicating greater symptom severity for pain interference, anxiety, depression, and social isolation. Ninety-five percent confidence intervals (CI) for domain means were calculated to aid interpretability; given small domain-level sample sizes (pain *n* = 8; other domains *n* = 9), these CIs are wide and reported as descriptive only.

Qualitative analysis was conducted on the five open-ended survey questions to provide contextualised insights complementing the quantitative data. Analysis of de-identified free-text responses was performed by a single researcher.

Reflexive thematic analysis, guided by Braun and Clarke’s six-phase framework [30], was used. Following familiarisation, 33 descriptive codes were generated, examined for latent meaning, and iteratively developed into broader themes.

An interpretivist stance underpinned the analysis, informed by the author’s professional background as an occupational therapist working in burn rehabilitation. A reflexive journal was used to document assumptions and decision-making to identify potential influences and support transparency. This helped ensure that analytical decisions remained grounded in participant perspectives rather than shaped by the author’s professional experiences.

Data saturation was not expected due to the small sample size and the exploratory nature of the service evaluation. Methodological rigor was supported using the reflexive journal and utilising a codebook to ensure transparency and trustworthiness within the analysis process.

No adverse events, infection-control incidents, or safety breaches related to participation were recorded during the study period.

## 3. Results

### 3.1. Quantitative Findings

Descriptive PROMIS scores suggested favourable outcomes across domains (outlined in Table 2). Lower scores indicated reduced symptom burden, while higher scores within physical function and emotional support reflected more positive perceived outcomes. Item-level non-responses are reported in Table 2 and were excluded from domain calculations.

#### 3.1.1. Pain Interference

Pain interference (*n* = 8) showed a moderate level overall. Mean = 2.5; SD = 1.10; 95% CI 1.58–3.42; range 1–4; mode 2.

8/9 (88.9%) participants responded to all pain items (see Table 2).

#### 3.1.2. Physical Function

Physical function (*n* = 9) was generally high. Mean = 4.66, SD = 0.80, 95% CI 4.05–5.27, range 1–5, median = 5, mode = 5.

All 9/9 participants responded to the physical function items.

#### 3.1.3. Emotional Distress—Anxiety Related

Emotional distress arising from anxiety was low to moderate (*n* = 9). Mean = 2.14; SD = 1.32; 95% CI 1.13–3.15; range 1–5; median = 2; mode = 1.

All 9/9 participants completed the anxiety items.

#### 3.1.4. Emotional Distress—Depression Related

Depression-related distress (*n* = 9) was generally low, with some individual variation. Mean = 1.84, SD = 1.05, 95% CI 1.03–2.65, range 1–4, median = 1, mode = 1.

All 9/9 participants completed the depression items.

#### 3.1.5. Emotional Support

Perceived emotional support (*n* = 9) was consistently high across participants. Mean = 4.50, SD = 0.80, 95% CI 3.89–5.11, range 3–5, median = 5, mode = 5.

All 9/9 participants completed these items.

#### 3.1.6. Social Isolation

Social isolation (*n* = 9) was generally low, with most responses clustered at the lower end despite some variation. Mean = 1.80, SD = 1.52, 95% CI 0.63–2.97, range 1–4, median = 1, mode = 1.

All 9/9 participants completed the social isolation items.

### 3.2. Qualitative Findings

Four overarching themes were revealed across participant responses: fostering human connection and psychological wellbeing; restoring autonomy, independence and confidence; physical and psychological preparation for recovery; and the importance of the rehabilitation environment.

#### 3.2.1. Fostering Human Connection and Psychological Wellbeing

Participants described sharing experiences as central to feeling understood, emphasizing the role of human connection in supporting psychological wellbeing. Many expressed that “meeting other burns patients” and “connecting with people who had experienced similar challenges” was particularly helpful. This suggests that these supportive interactions fostered a sense of belonging and helped reduce feelings of isolation by providing a psychologically safe “relaxed” environment that encouraged comfort, openness, and engagement.

Talking with others and hearing their stories were mentioned as specific benefits. Participants also described developing coping mechanisms and acceptance of their situation. As one participant reflected: “It made me realise it [the fire] was an accident and nothing I could have done could have prevented it.”

#### 3.2.2. Restoring Autonomy, Independence and Confidence

The option to participate in the group was identified as a motivational factor, supporting re-establishment of autonomy and daily routine. Participants reported that the group contributed to a sense of structure—for example, “[the group provided] me the morning routine.”

Others noted feeling “encouraged to participate” and were therefore more likely to get out of bed. Participants also described functional gains such as becoming “used to using hands again” and “gaining confidence,” indicating that the group supported both psychological and functional aspects of recovery.

#### 3.2.3. Importance of Environment for Rehabilitation

Participants indicated that a change in surroundings facilitated skill practice in contexts more reflective of everyday environments. The “change of environment” was frequently noted as helpful and motivating.

One participant described initial challenges due to “unfamiliar surroundings,” which were mitigated by “1:1 support to navigate the area,” contributing to “the beginnings of gaining confidence.” Many described no significant environmental challenges, reinforcing its role as a supportive context for rehabilitation.

#### 3.2.4. Physical and Psychological Preparation for Recovery

Participants described perceived functional improvements, including the development of motor skills through engagement in routine activities such as “using kitchen equipment,” which they felt supported their ability to manage daily living tasks.

The group also appeared to contribute to emotional readiness for discharge. One participant valued advance notice: “Being told in advance it was going to happen so that I wasn’t in the shower etc.” Another described “worries around the perception of others,” but still recognised the benefit of meeting peers “not possible otherwise.”

Several participants suggested that they would have liked the group to occur more frequently.

### 3.3. Triangulation

To align with survivor-centred narratives, quantitative and qualitative data were triangulated and summarised in Table 3. Quantitative results indicated high perceived physical function (mean = 4.66; 95% CI 4.05–5.27) and emotional support (mean = 4.50; 95% CI 3.89–5.11), with low reported social isolation (mean = 1.80; 95% CI 0.63–2.97), anxiety (mean = 2.14; 95% CI 1.13–3.15), and depression (mean = 1.84; 95% CI 1.03–2.65) (*n* = 9; item-level figures in Table 2).

Qualitative themes corroborated these descriptive trends, with participants describing the group as fostering human connection, supporting psychological wellbeing, restoring autonomy and confidence, facilitating rehabilitation, and preparing them for recovery.

Mapping findings to RE-AIM/MMR-RHS shows convergence under the Reach and Perceived Benefit dimensions. High physical function and emotional support scores aligned with narratives of regained confidence and reduced isolation, while open-ended accounts of barriers and facilitators explained variation in engagement.

Convergence was evident where PROMIS scores for emotional support, social isolation and physical function reflected participant descriptions of connection, confidence and functional engagement. Divergence emerged around pain interference where moderate interference scores did not always correspond with reduced motivation or participation. Both data sets provided complementary data, with quantitative data outlining a general pattern and qualitative narratives providing the contextual meaning and interpretation of these. Triangulation supports descriptive interpretation only and reflects survivor-reported experiences rather than intervention effects. Table 3 presents the mapping of quantitative indicators to qualitative themes, with explicit statistics and illustrative de-identified quotations to aid interpretation and transparency.

## 4. Discussion

To our knowledge, this is the first mixed methods service evaluation exploring a routine breakfast group activity in inpatient burn rehabilitation. Findings suggest that structured, peer-supported activities were perceived by participants as supportive of psychosocial wellbeing, social connection and functional engagement during inpatient rehabilitation [16].

Group-based approaches during inpatient burn care remain rare and under-researched, with most evidence focusing on community-based programmes or other clinical populations [16]. Building on Hill, O’Brien and Yurt’s [16] work, this evaluation contributes by describing a novel, integrated model of routine care that aims to support both physical and psychological recovery in a real-world inpatient setting. The breakfast group illustrates how peer-supported activity may foster holistic rehabilitation while potentially addressing structural challenges such as resource limitations, staffing constraints, and fragmented care pathways often encountered in inpatient environments [14].

This design may also be relevant to other complex clinical populations, such as spinal cord injury or severe mental health conditions, where isolation and reintegration challenges are similar. From a systems perspective, embedding group-based activity into routine care may offer a feasible and resource-efficient approach to supporting rehabilitation, aligning with health policy goals of patient-centred, efficient, and cost-effective delivery [14].

### 4.1. Reach

Reach was defined as the proportion of eligible patients who attended the breakfast group at least once. Due to the anonymous survey design and absence of linked attendance data, it was not possible to determine the characteristics of those who attended or did not attend to fully evaluate the intervention’s reach.

Although the survey data does not form part of the reach, completion rates provide important contextual information about engagement with the study and potential bias influencing interpretation.

#### Survey Completion

Survey completion was modest, with 9 of 36 invited participants completing the survey (25% completion rate). This response rate is consistent with known barriers in burn survivor research, including psychological distress, timing misalignment, fear of revisiting trauma, and social circumstances [31,32,33]. Notably, two individuals declined participation due to emotional distress and concerns about revisiting their injury experience, indicating possible bias towards more positive experiences among respondents.

Although modest, the sample included a range of ages and injury severities, suggesting partial representativeness despite low numbers. Nevertheless, participant narratives indicated strong acceptability among those who engaged, reinforcing the relevance of the activity despite limited reach. Future evaluations should explore strategies to improve reach, such as flexible recruitment timing and approaches tailored to survivors’ psychological readiness.

### 4.2. Perceived Benefit

Previous work highlights the value of holistic rehabilitation contributing to reduced isolation and strengthening psychological resilience [16,26]. Participant narratives in this evaluation similarly emphasised human connection as a central component of their experience.

Although protective isolation is prioritised in burn care to minimise infection risk and protect privacy, participants consistently valued opportunities to connect with peers, describing a strong need for shared experience and informal conversation. These findings suggest that infection-control priorities may inadvertently limit opportunities for therapeutic peer interaction, which participants perceived as important for psychological recovery. Future service development should consider how to balance biological safety with psychosocial needs.

Engagement in the breakfast group contributed to increased motivation, autonomy, and daily routine. Participants described the value of having control over daily decisions and engaging in purposeful activity, which supported a sense of continuity between pre- and post-injury identity. Meaningful daily activities were perceived as supporting psychological reassurance and functional readiness for life beyond hospitalisation [26,34].

Reduced distress and isolation scores converged with qualitative accounts of resilience, with structured peer interaction fostering coping mechanisms [6,7,8]. However, these findings reflect a single time point and cannot be attributed to the breakfast group alone.

Clinicians may consider structuring group-based activities to maximise psychosocial benefit while maintaining infection-control and safety standards inherent to inpatient care.

### 4.3. Adoption

The evaluation included only individuals who had already opted into the activity. Their pre-existing positive perceptions may have influenced both engagement and reported experiences [24,35]. This introduces participation bias, as the evaluation does not capture the perspectives of those who declined or were unable to attend.

While this focus aligns with the evaluation’s aims, concentrating exclusively on survivor perspectives omits staff and organisational viewpoints, limiting understanding of factors such as perceived benefit, logistical barriers, or institutional constraints that shape adoption. Without insight into these experiences, the adaptations required to extend reach remain uncertain [35].

Future evaluations should adopt a multi-stakeholder approach, incorporating staff perspectives to capture organisational and clinical insights that may influence adoption and sustainability.

### 4.4. Implementation

Although not formally measured, qualitative data suggested several practical implementation insights that may support other burn care providers in adopting similar group-based interventions. Participants consistently highlighted the value of the non-ward environment, which appeared to enhance motivation, confidence, and engagement by providing a more typical daily-living context. This contributed to the group being experienced as both “relaxed” and “structured.” Flexible one-to-one support was important for individuals who felt anxious or uncertain in unfamiliar surroundings, helping to build confidence and maintain a “supportive” atmosphere. Delivering the group within existing staffing was feasible, and predictable structure and advance notice were appreciated by participants. These practical observations may assist other burn services in embedding routine, occupation-based group activity within inpatient rehabilitation. Several contextual and structural challenges were reported that may impact broader implementation. Timing and scheduling emerged as critical factors, with some participants noting the need for physical and psychological readiness to support participation. This highlights the importance of flexible scheduling and clear communication to enhance accessibility.

Infection-control requirements and ward routines are unique constraints that must be addressed when scaling or replicating the activity. Staffing and physical space were also noted as potential barriers. Increasing frequency, as suggested by participants, would require careful consideration of these constraints. Adequate staffing and integration within rehabilitation routines are important for supporting individual recovery [16,26,34].

Fidelity to the activity model was not formally assessed, representing a limitation for understanding consistency of delivery.

### 4.5. Maintenance

Participants’ suggestions to increase the frequency of the activity indicate strong perceived benefits and potential for integration into routine care. Despite variation in survey timing, participants consistently recalled positive experiences, suggesting a meaningful contribution to their recovery journey.

For sustained delivery, feasibility issues, including staff capacity, infection-control requirements, and ward routines, must be addressed. Prospective longitudinal research is needed to assess the durability of perceived benefits and to evaluate long-term integration within inpatient rehabilitation pathways.

### 4.6. Limitations

This service evaluation has several limitations that should be considered when interpreting the findings.

The small sample size (*n* = 9) and low response rate (25%) limit the breadth of perspectives captured and introduce potential selection bias, as individuals with more positive experiences may have been more likely to participate. Furthermore, although attendance was routinely documented, survey anonymity prevented linking individual responses to attendance frequency; as a result, it was not possible to determine if respondents were first-time or recurring attendees.

The absence of demographic data, including age, gender, ethnicity, injury characteristics, and inpatient/outpatient status, restricts understanding of representativeness and limits subgroup interpretation. No power calculation was undertaken, consistent with the exploratory nature of the evaluation, and quantitative findings are descriptive only.

The evaluation relied on a single time-point survey, meaning that PROMIS scores reflect participants’ experiences at the time of completion and cannot be attributed to the breakfast group. The absence of participant checking, due to the survey format, is acknowledged as a limitation that may have constrained validation of qualitative interpretations. The absence of baseline or follow-up data prevents assessment of change over time. Therefore, findings cannot indicate change or causation and should be interpreted as survivor-reported perceptions only.

Qualitative analysis was conducted by a single researcher, which may have influenced interpretation. However, reflexive thematic analysis acknowledges the researcher’s interpretive role, and transparency was supported through a reflexive journal and codebook. Data saturation was not expected due to the small sample and exploratory purpose of the evaluation.

Fidelity to the breakfast group model was not formally assessed, limiting understanding of consistency in delivery. Additionally, resilience and post-traumatic growth were not measured, despite their relevance to burn recovery, and should be considered in future evaluations.

Finally, the variation in the time elapsed between group attendance and survey completion may have introduced recall bias.

## 5. Conclusions

This service evaluation demonstrated that a peer-supported breakfast group was acceptable to adult burn inpatients and offered perceived psychological and social benefit when delivered as part of routine inpatient burn rehabilitation. Triangulated qualitative and quantitative findings indicated that participants found human connection, normalisation, and emotional wellbeing through the group in their recoveries. These findings highlight the potential value of integrating structured, peer-supported activity into inpatient burn care, particularly in addressing psychosocial needs.

However, limitations in survey response rates, anonymous data collection, and small sample size restrict the generalisability of results and limit deeper analysis of who did and did not engage. Future evaluations should incorporate demographic data, pre/post measurements and approaches tailored to survivors’ psychological readiness to strengthen understanding of reach and optimise engagement, as well as consideration of multi-stakeholder perspectives to evaluate the role of group-based activity more comprehensively within burn rehabilitation. Despite these constraints, the evaluation provides early evidence that peer-supported group activity may enhance inpatient burn recovery and warrants further exploration in larger, more robust studies.

## Figures and Tables

**Table 1:** Inclusion and Exclusion Criteria.

Inclusion Criteria	Exclusion Criteria
Adults (18+) with a burn or scald injury who were inpatients at the burns unit. Attended the inpatient breakfast group between September 2022 and February 2025. Able to communicate in English language. Possess mental capacity to consent.	Individuals with non-burn injuries, including medical skin loss conditions. Survivors who did not attend the breakfast group during their inpatient stay. Aged under 18 Unable to communicate in English language. Lacking mental capacity to consent.

**Table 2:** Summary of Participant Scores Across PROMIS Domains.

PROMIS Domain	Sample Size (*n =* Respondents)	Mean	Median	Mode	Standard Deviation (SD)	Range (Min–Max)	Interquartile Range (IQR) (Q1–Q3)	Confidence Interval (CI) Lower (95%)	Confidence Interval (CI) Upper (95%)
Pain Interference	8	2.5	2	2	1.10	1–4	1.25	1.58	3.42
Physical Function	9	4.66	5	5	0.80	1–5	0	4.05	5.27
Anxiety-Related Distress	9	2.14	2	1	1.32	1–5	2	1.13	3.15
Depression-Related Distress	9	1.84	1	1	1.05	1–4	2	1.03	2.65
Emotional Support	9	4.5	5	5	0.80	3–5	1	3.89	5.11
Social Isolation	9	1.8	1	1	1.52	1–4	2	0.63	2.97

Item-level respondent counts: Pain Interference *n* = 8; all other PROMIS domains *n* = 9.

**Table 3:** Triangulation Interpretation Summary.

Interpretation (Triangulation Insight)	Quantitative Finding(s)	Qualitative Theme(s)	Mapped RE-AIM Dimension
Participants described pain as having a mild but significant interference on social activities, but it did not seem to impact engagement in the breakfast group.	Mild pain interference (mean = 2.5)	N/A (quantitative only) *	Effectiveness
Survivors described improved function and confidence through routine engagement (e.g., using hands, kitchen tasks), which is reflected in high physical function scores.	High physical function (mean = 4.66)	Restoring Autonomy, Independence and Confidence	Effectiveness
Consistently high scores for emotional support align with survivors’ descriptions of a relaxed, safe setting that enabled openness and mutual understanding.	High emotional support (mean = 4.5)	Fostering Human Connection and Psychological Wellbeing	Effectiveness
Participants’ narratives aligned with low distress scores, suggesting perceived emotional benefit; however, no causal relationship can be inferred.	Low Anxiety (mean = 2.14) and Depression-related (mean = 1.84) distress	Psychological Preparation for Recovery	Effectiveness
Quantitative data suggests minimal social isolation supported by narratives about feelings of connectedness and understanding indicating the group fostering peer interaction and reducing loneliness.	Low social isolation (mean = 1.8)	Fostering Human Connection and Psychological Wellbeing	Effectiveness
Participants reported that tailored staff support and structured settings made the group more accessible and welcoming.	Not applicable as reflected in qualitative data only.	Importance of Environment for Rehabilitation	Adoption, Implementation

* N/A = Not applicable; quantitative data only.

## Data Availability

Due to the sensitive nature of clinical service evaluation data, datasets are not publicly available. Data supporting the findings of this study may be available from the author upon reasonable request.

## Supplementary Materials

The following supporting information can be downloaded at: https://www.mdpi.com/article/10.3390/ebj7030040/s1, File S1: participant survey.
