# Supplementary material for: A Mixed Methods Service Evaluation of a Peer-Supported Breakfast Group in Adult Inpatient Burn Rehabilitation
Source: Eur Burn J. 2026 Jul 17;7(3):40. doi: 10.3390/ebj7030040 (PMC13397859; doi:10.3390/ebj7030040)
Supplement: Supplementary file 1 [file ebj-07-00040-s001.zip › ebj-4380625-supplementary.pdf]

PARTICIPANT SURVEY
Exploring the impact of the group therapy intervention 'breakfast group' on inpatient adult burn survivors: a mixed methods service evaluation.

Thank you for taking part in the Breakfast Club service evaluation.

Your feedback is vital in helping us improve services for burn survivors. Participation is **entirely voluntary**, and you are free to skip any questions you do not wish to answer. You can withdraw from the survey at any time without any impact on your care. If you wish to withdraw your data after completion, please contact the researcher directly.
To ensure we can identify and remove your data, please keep your unique ID safe as you will need it to withdraw.

Please be aware that some questions may prompt emotional distress, particularly regarding trauma or body image. You may skip any questions that are uncomfortable and contact details for psychological support services are available if needed. If you have any concerns, the researcher (Lottie Armitage lottie2.armitage@live.uwe.ac.uk) is available to assist.

All responses are confidential, and no personally identifiable information will be linked to the data. A unique ID will be used to protect your anonymity. If any data is deemed to be identifiable (such as demographic information), it will be redacted prior to analysis.

If you have any questions or need help, please feel free to contact us.

Thank you again for your participation.

PART 1

**1. What aspects of the breakfast group did you find most helpful or motivating?**
**2. What challenges or barriers did you face in participating in the breakfast group?**

**3. Please describe any specific benefits you have experienced from participating in the breakfast group**
**4. Can you share any challenges you encountered while participating in the breakfast group?**
**5. What changes, if any, would you suggest improving the breakfast group sessions**

PART 2 OVERLEAF

PART 2

Please respond to each item by marking one box per row.

**During the breakfast group sessions attended…**	**Not at all**	**A little bit**	**Somewhat**	**Quite a bit**	**Very much**
How much did pain interfere with your enjoyment of life?	☐ 1	☐ 2	☐ 3	☐ 4	☐ 5
How much did pain interfere with your day to day activities?	☐ 1	☐ 2	☐ 3	☐ 4	☐ 5
How much did pain interfere with your ability to participate in social activities?	☐ 1	☐ 2	☐ 3	☐ 4	☐ 5
	**Without any difficulty**	**With a little Difficulty**	**With some difficulty**	**With much difficulty**	**Unable to do**
Were you able to cut your food using eating utensils?	☐ 5	☐ 4	☐ 3	☐ 2	☐ 1
Were you able to hold a plate full of food?	☐ 5	☐ 4	☐ 3	☐ 2	☐ 1
Were you able to pour liquid from a bottle into a glass?	☐ 5	☐ 4	☐ 3	☐ 2	☐ 1
Were you able to lift a full cup or glass to your mouth?	☐ 5	☐ 4	☐ 3	☐ 2	☐ 1
Were you able to open a new milk carton?	☐ 5	☐ 4	☐ 3	☐ 2	☐ 1
	**Never**	**Rarely**	**Sometimes**	**Often**	**Always**
I felt fearful	☐ 1	☐ 2	☐ 3	☐ 4	☐ 5
I felt worried	☐ 1	☐ 2	☐ 3	☐ 4	☐ 5
I worried about other people's reactions to me	☐ 1	☐ 2	☐ 3	☐ 4	☐ 5
	**Never**	**Rarely**	**Sometimes**	**Often**	**Always**
I withdrew from other people	☐ 1	☐ 2	☐ 3	☐ 4	☐ 5
I felt that I was not as good as other people	☐ 1	☐ 2	☐ 3	☐ 4	☐ 5

I felt lonely	☐<br>1	☐<br>2	☐<br>3-	☐<br>4	☐<br>5
I felt ignored by people	☐<br>1	☐<br>2	☐<br>3	☐<br>4	☐<br>5
	**Never**	**Rarely**	**Sometimes**	**Often**	**Always**
I had people who I could talk to about my health	☐<br>1	☐<br>2	☐<br>3	☐<br>4	☐<br>5
I had someone who understood my problems	☐<br>1	☐<br>2	☐<br>3	☐<br>4	☐<br>5
I felt eel there were people who really understand me	☐<br>1	☐<br>2	☐<br>3	☐<br>4	☐<br>5
I felt isolated even when I am not alone	☐<br>1	☐<br>2	☐<br>3	☐<br>4	☐<br>5
I felt that people avoid talking to me	☐<br>1	☐<br>2	☐<br>3	☐<br>4	☐<br>5
I felt detached from other people	☐<br>1	☐<br>2	☐<br>3	☐<br>4	☐<br>5

Thank you for your participation.

Please remember when sending this back to return with the signed consent form otherwise the survey will not be able to be used and be destroyed.

If you feel you need additional support please contact your burns team who can direct you or the Adult Burn Support which is run in conjunction with Dans Fund for Burns and is available at: https://adultburnsupportuk.org/
